# Supplementary material for: The interactome of intact mitochondria by cross-linking mass spectrometry provides evidence for coexisting respiratory supercomplexes
Source: Mol Cell Proteomics. 2017 Dec 8;17(2):216–32. doi: 10.1074/mcp.RA117.000470 (PMC5795388; doi:10.1074/mcp.RA117.000470)
Supplement: Supplemental Data [file supp_RA117.000470_133922_0_supp_23978_fzffwf.zip › spectra_annotation/mito_spectra_annotation/437-1-5-1-1-1.pdf]

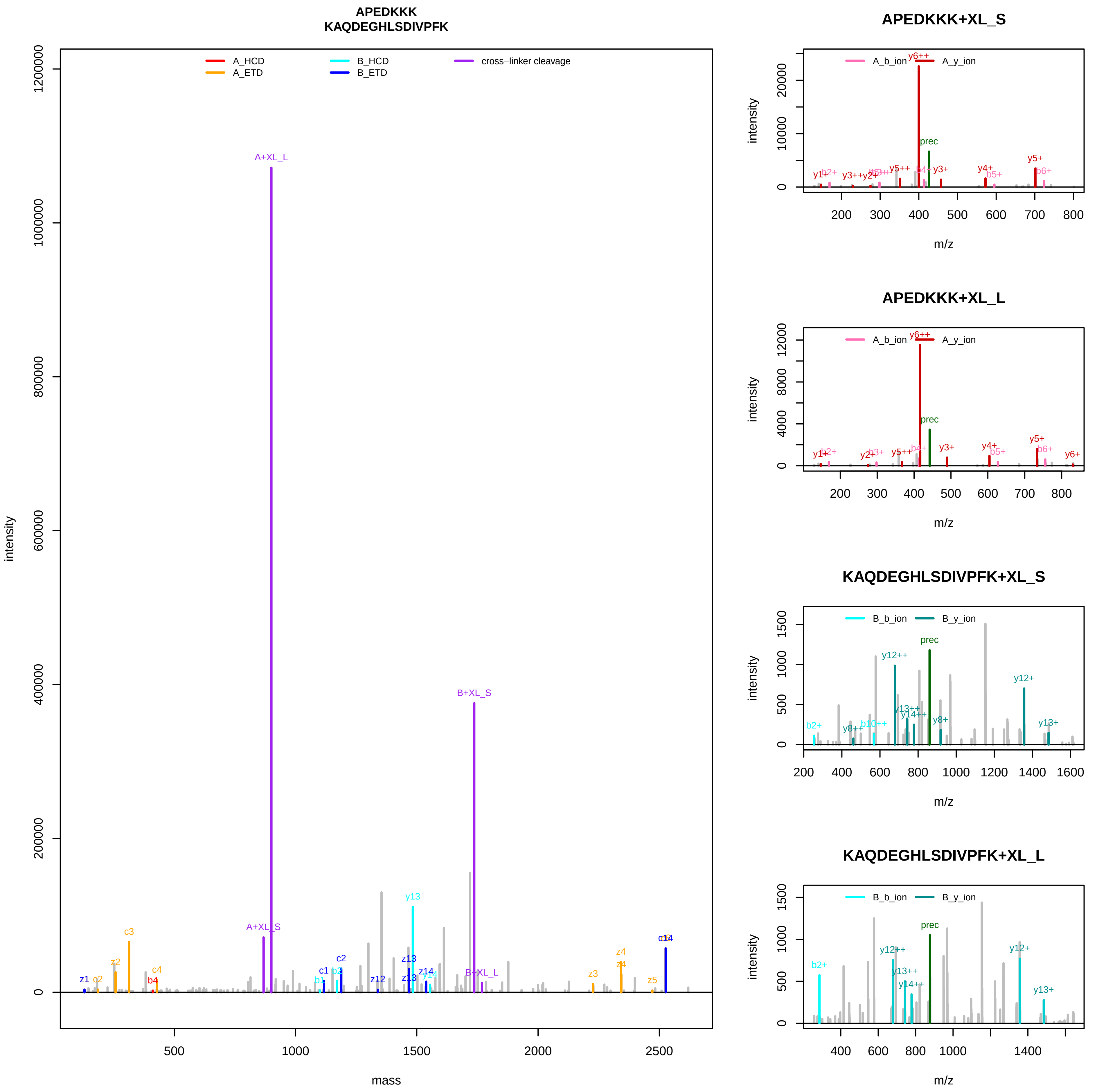

APEDKKK
KAQDEGHLSDIVPFK
A_HCD
A_ETD
B_HCD
B_ETD
cross−linker cleavage
A+XL_L
A+XL_S
B+XL_S
B+XL_L
y13
c3
z2
c4
b4
z1
c2
c1
b2
b1
z12
z13
z14
y14
z3
z4
z5
c14
intensity
mass
APEDKKK+XL_S
APEDKKK+XL_L
KAQDEGHLSDIVPFK+XL_S
KAQDEGHLSDIVPFK+XL_L
A_b_ion
A_y_ion
B_b_ion
B_y_ion
prec
y6++
y5+
y4+
y3+
y1+
b2+
b5+
b6+
y12++
y12+
y13+
y13++
y14++
y8+
y8++
b10++
m/z
